# Supplementary material for: Disentangling metabolic functions of bacteria in the honey bee gut
Source: PLoS Biol. 2017 Dec 12;15(12):e2003467. doi: 10.1371/journal.pbio.2003467 (PMC5726620; doi:10.1371/journal.pbio.2003467)
Supplement: S8 Data — (ZIP) [file pbio.2003467.s008.zip › Z-score_plots/983 microbial substrate Quercetin 3-O-[beta-D-xylosyl-(1-2)-beta-D-glucoside].pdf]

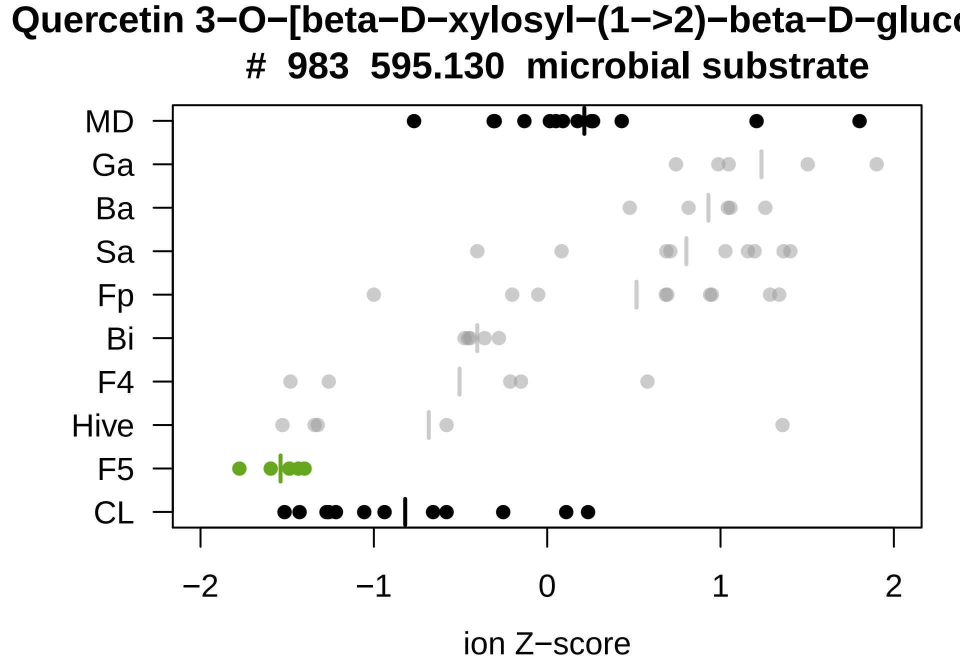

Quercetin 3−O−[beta−D−xylosyl−(1−>2)−beta−D−gluco
983 595.130 microbial substrate
MD
Ga
Ba
Sa
Fp
Bi
F4
Hive
F5
CL
−2
−1
0
1
2
ion Z−score
